# Collaboration between a human group and artificial intelligence can improve prediction of multiple sclerosis course: a proof-of-principle study

**DOI:** 10.12688/f1000research.13114.2

**Published:** 2018-08-01

**Authors:** Andrea Tacchella, Silvia Romano, Michela Ferraldeschi, Marco Salvetti, Andrea Zaccaria, Andrea Crisanti, Francesca Grassi

**Affiliations:** 1 Institute for Complex Systems, National Research Council - UOS Sapienza, Rome, 00185, Italy; 2 Center for Experimental Neurological Therapies (CENTERS), Dept. of Neurosciences, Mental Health and Sensory Organs, Sapienza University of Rome, Rome, 00189, Italy; 3 IRCCS Neuromed , Istituto Neurologico Mediterraneo, Pozzilli, 86077, Italy; 4 Department of Physics, Sapienza University of Rome, Rome, 00185, Italy; 5 Institute Pasteur-Cenci Bolognetti Foundation, Dept. Physiology and Pharmacology, Sapienza University of Rome, Rome, 00185, Italy

**Keywords:** Multiple sclerosis, Machine learning, Random Forest, collective intelligence, Hybrid predictions, Crowdsourcing

## Abstract

**Background:** Multiple sclerosis has an extremely variable natural course. In most patients, disease starts with a relapsing-remitting (RR) phase, which proceeds to a secondary progressive (SP) form. The duration of the RR phase is hard to predict, and to date predictions on the rate of disease progression remain suboptimal. This limits the opportunity to tailor therapy on an individual patient's prognosis, in spite of the choice of several therapeutic options.

Approaches to improve clinical decisions, such as collective intelligence of human groups and machine learning algorithms are widely investigated.

**Methods:** Medical students and a machine learning algorithm predicted the course of disease on the basis of randomly chosen clinical records of patients that attended at the Multiple Sclerosis service of Sant'Andrea hospital in Rome.

**Results:** A significant improvement of predictive ability was obtained when predictions were combined with a weight that depends on the consistence of human (or algorithm) forecasts on a given clinical record.

**Conclusions:** In this work we present proof-of-principle that human-machine hybrid predictions yield better prognoses than machine learning algorithms or groups of humans alone. To strengthen and generalize this preliminary result, we propose a crowdsourcing initiative to collect prognoses by physicians on an expanded set of patients.

## Introduction

The natural course of multiple sclerosis (MS) is extremely variable, ranging from extremely mild to very aggressive forms. Most patients experience an initial relapsing-remitting (RR) phase, in which symptoms appear and fade. Eventually, remissions fail and the disease proceeds to a secondary progressive (SP) form, leading to incremental disability. The palette of disease-modifying treatments is becoming relatively large, in principle opening the possibility to tailor the therapy to meet the specific needs of each patient. Unfortunately, the accuracy of parameters to predict the rate of disease progression remains suboptimal.

Being all the above therapies preventive, in the absence of exact prognostic indicators we have to accept that a proportion of patients is either under- or over-treated. This is a serious concern as the disease can be severely disabling, and some of the available therapies can lead to adverse events that can be worse than the disease itself. Thus, the possibility to formulate a prognosis as exact as possible is becoming increasingly appealing.

In the clinics, as in any other fields of human knowledge, innovative approaches based on machine learning and collective reasoning methods are used in an attempt to succeed where traditional methods of forecasting failed. Machine learning algorithms catch complex relations among existing data to an extent beyond standard regression models. Good performances have been obtained for the diagnosis of Parkinson's disease and the prognosis of disease progression in amyotrophic lateral sclerosis ( Dinov *et al.*, 2016; Küffner *et al.*, 2015). For MS, machine learning algorithms can correctly classify disease course in about 70% of cases of both clinically definite MS and of clinically isolated syndrome ( Fiorini *et al.*, 2015; Wottschel *et al.*, 2014; Zhao *et al.*, 2017), a good result that still requires improvement to become of clinical value.

Through collective reasoning, or collective intelligence, groups of lay people may perform as well as experts. In principle, the larger the group, the higher the prediction accuracy (see for review Ponsonby & Mattingly, 2015), which led to the development of several crowdsourcing initiatives. Possibly, the forerunner was FOLDIT study on protein folding ( Cooper *et al.*, 2010), but crowdsourcing has been exploited also for diagnostic purposes in pathologies, such as breast cancer ( Candido dos Reis *et al.*, 2015), skin cancer ( King *et al.*, 2013) or ophtalmology ( Wang *et al.*, 2016). However, when expert people are involved, even small groups can outperform the best among them, at least when a yes/no answer to well-defined diagnostic questions is requested based on radiographic/histological images, ( Kurvers *et al.*, 2016; Sonabend *et al.*, 2017; Wolf *et al.*, 2015). Studies with medical students show that working in pairs, either interacting while responding ( Hautz *et al.*, 2015) or aggregating responses *ex post* ( Kämmer *et al.*, 2017), ameliorates diagnostic ability, with further improvements when group size increases ( Hautz *et al.*, 2015; Kämmer *et al.*, 2017), in line with the core idea of Collective intelligence. Similar results have been obtained also for prognoses on critically ill patients ( Poses *et al.*, 1990)

Combination of human and machine predictions into hybrid forecasts exploits human intuitive reasoning and computer classification capabilities, potentially boosting both. Indeed, at least in the case of predicting the course of actions in American football games within the frame of prediction markets, hybrid groups performed better than either humans or computers. ( Nagar & Malone, 2011). In this paper, we report the promising results of a preliminary study on the combination of predictions made by humans with those of a machine learning algorithm on the progression of multiple sclerosis in a set of patients. Both agents (humans and computers) considered clinical data typically available to neurologists during routine visits. Magnetic Resonance Imaging (MRI) data were not included as more clinical than radiological exams are routinely performed (on average 3 visits per year *vs.* 1 MRI). Moreover, images, acquired and analysed at specialized centres can improve the algorithm performance ( Zhao *et al.*, 2017), but in real world imaging data usually lack the standardization required for analysis, for instance in term of head position reproducibility ( Weinstock-Guttman *et al.*, 2018), and research-grade image analysis is not routinely performed. Conversely, clinical data have recently been shown to have good predictive value ( Goodin *et al.*, 2018). Machine learning and collective intelligence performed almost equally well, but their combination yielded a small, yet statistically significant, improvement in the reliability of the forecasts on disease evolution over different time periods.

These results indicate that it is worth deepening the study of human and machine clinical predictions, as well as the potentiality of hybrid predictions, for which we propose a crowdsourcing approach on a platform specifically designed for this analysis ( *DiagnoShare*).

## Methods and results

### Dataset structure

Our dataset is composed by clinical records gathered during 527 visits of 84 outpatients followed at the Multiple Sclerosis service of Sant’Andrea hospital in Rome. All patients had clinically definite MS in the RR stage at the time of the visit(s) included in the database and transitioned to the SP phase at some time point. Parameters evaluated during each neurological visit are listed in Supplementary Table 1. Numerical values were provided for each parameter, referring to age, time to complete a task, clinical score or presence/absence of each symptom. For each visit, we noted if the patient was in RR or SP stage after 180, 360 and 720 days, so that predictions could be compared with the *true* progression of disease in each patient, reported in Supplementary File: TrueOutcomes.xlsx, where 0 means "still in RR phase", 1 indicates "transitioned to SP phase". Notice that several patients reached the SP MS stage after the last visit included in the database, so that the number (percentage of entries) of "1" records is 65 (12.3%), 125 (23.7%), and 211 (40.0%) at 180, 360, and 720 days, respectively. Data potentially revealing the identity of the patients was removed from the shared database.

### Ethics

Use of database for research purposes was authorized by the Ethical committee of Sapienza University (Authorization n. 4254_2016, dated November 2, 2016).

### Classification with machine learning

Having a correctly labelled dataset ( Supplementary Table 1), in which each entry is associated to the outcome, we used the Random forest supervised approach to classification ( Breiman, 2001; Liaw & Weiner, 2002), using the *Scikit-learn* toolbox version 0.16.1.

To benchmark the performance of the trained models, we used a modified *leave-one-out approach*. Since data was limited (a set of 527 records), and not independent, as it had been obtained from 84 patients, with a simple random *leave-one-out* the training set would be composed of many correlated same-patient data. Even worse, some of the data from patients present in the training set would be used to validate the model in the benchmarking stage. As a consequence, the model would overfit the training data, misleadingly showing very good performance. Being presented with many data from the same patient, the model optimizes its ability in recognizing patients themselves, through their highly correlated clinical variables.

To avoid these problems, we used a modified *leave-one-out* approach, training the algorithm with the following rules:

1. We excluded all visits from one patient from the dataset
2. We built 50 training sets, each composed by 83 records, taking care to include only one clinical record (randomly chosen) for every remaining patient
3. We trained 50 Random Forest models, one for each training set.
4. We computed the probability of the transition from RR to SP by averaging the predictions of the 50 models on all the visits of the excluded patient. Predictions consisted in scores from 0 (Extremely unlikely) to 1 (Highly probable).

We repeated the procedure for the 84 patients, obtaining an estimation of the probability of the RR to SP transition for each of the 527 clinical records. Three different prediction delays were considered, namely 180, 360 and 720 days. Results obtained are presented in Supplementary File: RF_Predictions.xlsx. The performance of the model was estimated by the Area Under the " *Receiver Operating Characteristic*" (ROC) Curve (AUC) computed on all the 527 examples. The AUC values obtained are shown in Table 1.

**Table 1.** Predictions on disease course by different agents.

Agent	180 days	360 days	720 days
Random Forest	0.710	0.670	0.679
*Singles (n=42)*	*0.57 ± 0.15*	*0.57 ± 0.11*	*0.57 ± 0.10*
*Pairs*	*0.68*	*0.65*	*0.65*
*Group*	*0.703*	*0.667*	*0.666*
Hybrid predictions	0.725 *	0.694 *	0.696 *

For each clinical record, the indicated agents evaluated the probability that disease evolved from the RR to the SP phase after 180, 360 or 720 days. Data represent the AUC values obtained for each method. *: P<0.001 when compared to *Group* or Random Forest values at the same time points.

### Human predictions

Forty-two medical students in the final two years of their course (Sapienza University, Rome Italy, based within Sant'Andrea hospital), volunteered to participate in the task. All were familiar with clinical records in general, and were instructed on the meaning of each entry present in the medical records of MS patients. This part of the study was approved by the Ethical Committee of the Department of Physiology and Pharmacology, Sapienza University on July 13, 2017.

For adequate comparison with computer predictions, students evaluated 50 medical records, collected in a questionnaire, randomly extracted from the same dataset used for machine learning and estimated the probability that the patient would progress to the SP phase within 180, 360 and 720 days. Scores were from 0 (Extremely unlikely) to 5 (Highly probable). Predictions (see Supplementary file Student_Predictions.xlsx) were analysed, using the AUC.

On average, each clinical record was evaluated by 4 of the 42 students.

Predictions were less accurate than those proposed by machine learning ( Table 1). Standard deviation was larger for the 180 day time point, indicating that opinions on the long-term evolution of the disease are more widely shared, although they are not more precise. To evaluate the impact of collective intelligence, we measured the performance of *Pairs*, considering all visits evaluated by at least two individual students, randomly selecting only 2 scores when more were available. The prognoses were averaged before computing the AUC, which showed a marked increase ( Table 1). Aggregation of all singles ( *Group*) yielded a further small increase in the performance of the forecasting ( Table 1), which almost equalled that of random forest algorithm.

### Hybrid predictions

We next integrated human and computer predictions into a hybrid prediction, which combines human clinical reasoning with the classification approach of machine learning algorithms. These different "ways of reasoning" possibly lead to quite divergent predictions on individual cases, a complementarity that should be exploited taking the difference into account when creating hybrid predictions.

The simplest approach to aggregate forecast is performing a linear or weighted average of the predictions released by humans or computer. For each clinical record at a given time point (180, 360 and 720 days), the final forecast by either agent is the average of “unitary predictions” given by several individuals or decision trees. If “unitary predictions” of one agent are highly concordant, it means that the prediction is quite obvious for the agent, suggesting that it is probably correct. We therefore ranked forecasts on clinical records in order of concordance of “unitary predictions”, for the two agents separately. Then, a normalized ranking was assigned, ranging from 1 for the most consistent predictions to 0 for the most scattered and ranks were squared to emphasize the contribution of the most consistent agent. The hybrid prediction score for each clinical record was then obtained by summing the two squared ranks.

Note that a linear combination of rankings resulted in a worse performance of hybrid predictions, as the information about the most consistent prediction between the two agents was be lost. A similarly degraded performance was observed when predictions were not ranked.

Since our dataset is relatively small, as is the number of students that evaluated the clinical records, we used a bootstrap procedure to evaluate the statistical significance of the improvement. The bootstrap ( Efron & Tibshirani, 1994; Felsenstein, 1985) consists in random sampling of the dataset that allows the estimation of confidence intervals.

**Figure 1.**
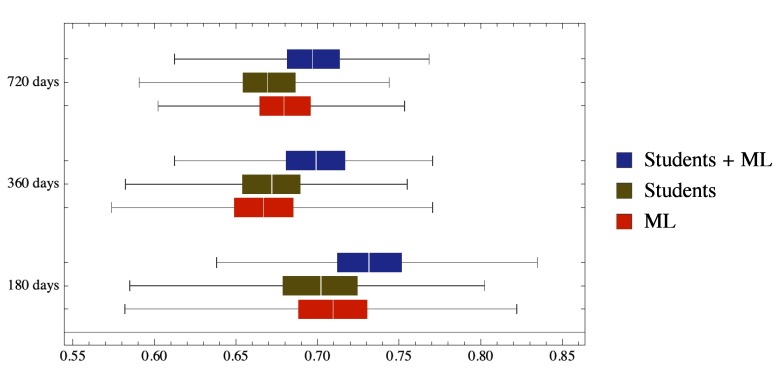
Hybrid Students – Machine Learning predictions outperform both human group and computer alone. The box plot shows the distribution of the AUC obtained from the bootstrap. In particular, the colored boxes correspond to quartiles, while the lines show the full range of the generated AUCs.

As shown in Table 1 and Figure 1, hybrid predictions yielded a small but statistically significant (P<0.001) improvement in the prediction of disease course in time. Significance was evaluated from confidence limits using standard methods ( Altman & Bland, 2011).

True outcome of patients, indexed as clinical records More than one clinical record is pertinent to each patient. T_180, T_360, T_720: clinical conditions of the patient 180, 360 and 720 days after the visit in which clinical record was obtained. 0: still in RR phase; 1: transitioned to SP phase. Click here for additional data file. Copyright: © 2018 Tacchella A et al. 2018 Data associated with the article are available under the terms of the Creative Commons Zero "No rights reserved" data waiver (CC0 1.0 Public domain dedication).

Predictions on individual clinical records made by medical students Each student worked on a questionnaire (lines labelled "questionnaire", column B.) listing 50 clinical reports (lines labelled "Clinical report N", columns B to AY) and made a prediction on the probability of RR –to–SP transition within 180, 360 and 720 days (lines labelled Prediction @ 180, 360, 720, columns B to AY)
The numbering of Clinical reports is the same used in Dataset 1. Click here for additional data file. Copyright: © 2018 Tacchella A et al. 2018 Data associated with the article are available under the terms of the Creative Commons Zero "No rights reserved" data waiver (CC0 1.0 Public domain dedication).

Predictions on individual clinical records made by a Random Forest algorithm Score_180, Score_360, Score_720: Probability that the patient will transition to SP phase within 180, 360 and 720 days after the visit in which clinical record was obtained. The numbering of Clinical reports is the same used in Dataset 1. Click here for additional data file. Copyright: © 2018 Tacchella A et al. 2018 Data associated with the article are available under the terms of the Creative Commons Zero "No rights reserved" data waiver (CC0 1.0 Public domain dedication).

## Discussion

A number of studies have investigated the possibility to increase the appropriateness of clinical decisions through collective intelligence of human groups (for instance, Kurvers *et al.*, 2016; Sonabend *et al.*, 2017; Wolf *et al.*, 2015) or machine learning algorithms. The latter approach has been used in a great variety of tasks, and its value in the medical realm is possibly overstated ( Chen & Asch, 2017). However, machine learning methods performed well for prognostic predictions ( Küffner *et al.*, 2015; Zhao *et al.*, 2017). In particular, the Random forest approach provided good predictions on ALS course ( Küffner *et al.*, 2015).

In this work we present proof-of-principle that human-machine hybrid predictions attain prognostic ability above that of machine learning algorithms and groups of humans alone.

The duration of the RR phase before its shift into progression has always been difficult to predict, and possibly the random occurrence of relapses ( Bordi *et al.*, 2013) contributes to the lack of univocal indicators. No approach, no matter how good, can yield certainty when cause-effect relations are unknown. Thus, our aim has been to obtain predictions on the probability that MS patients in the RR phase will convert to a SP form within a certain time frame. Predictions on the course of real patients were provided by medical students and a random forest algorithm. A significant improvement of predictive ability was obtained when predictions were combined in a non-linear manner, with a weight that depends on the consistence of human (or algorithm) forecasts on a given clinical record.

This result can be considered in agreement with several studies on different medical issues showing that predictor's confidence correlates very well with the correctness of the prediction ( Detsky *et al.*, 2017; Hautz *et al.*, 2015; Kämmer *et al.*, 2017; Kurvers *et al.*, 2016). Indeed, the concordance of different members of a given group (students or runs of the random forest model) can be taken as indicating that the agent is "sure" of the forecast. Further work investigating the best ways to combine predictions of different agents is ongoing.

In spite of the relatively basic machine learning technique used, the small number of students involved and their limited clinical knowledge, this work suggests that hybrid predictions can be useful to improve the prognosis of MS course. A deeper study is therefore of interest, to evaluate how general this conclusion is. To recruit more and more skilled humans, we propose a crowdsourcing initiative called *DiagnoShare* that is being advertised among physicians.

A reliable tool to predict MS progression can be of aid to clinicians to tailor therapy to each patient, but also in clinical trials, to evaluate whether drugs modify the estimated outcome of each enrolled patient, as proposed for ALS ( Küffner *et al.*, 2015).

In the long run, it is possible that further developments in our ability to combine collective reasoning and machine predictions will have a profound impact also on the organization and management of medical care, particularly in hospital settings.

## Data availability

The data referenced by this article are under copyright with the following copyright statement: Copyright: © 2018 Tacchella A et al.

Data associated with the article are available under the terms of the Creative Commons Zero "No rights reserved" data waiver (CC0 1.0 Public domain dedication). 

Dataset 1: *True outcome of patients, indexed as clinical records*. More than one clinical record is pertinent to each patient. T_180, T_360, T_720: clinical conditions of the patient 180, 360 and 720 days after the visit in which clinical record was obtained. 0: still in RR phase; 1: transitioned to SP phase. DOI: 10.5256/f1000research.13114.d188355 ( Tacchella *et al.*, 2017a)

Dataset 2: *Predictions on individual clinical records made by medical students.* Each student worked on a questionnaire (lines labelled "questionnaire", column B.) listing 50 clinical reports (lines labelled "Clinical report N", columns B to AY) and made a prediction on the probability of RR –to–SP transition within 180, 360 and 720 days (lines labelled Prediction @ 180, 360, 720, columns B to AY)

The numbering of Clinical reports is the same used in Dataset 1. DOI: 10.5256/f1000research.13114.d188356 ( Tacchella *et al.*, 2017b)

Dataset 3: *Predictions on individual clinical records made by a Random Forest algorithm*. Score_180, Score_360, Score_720: Probability that the patient will transition to SP phase within 180, 360 and 720 days after the visit in which clinical record was obtained. The numbering of Clinical reports is the same used in Dataset 1. DOI: 10.5256/f1000research.13114.d188357 ( Tacchella *et al.*, 2017c)

## Supplementary material

Supplementary Table 1: Parameters evaluated for each patient and included in clinical records.
