# Supplementary material for: Collaboration between a human group and artificial intelligence can improve prediction of multiple sclerosis course: a proof-of-principle study
Source: F1000Res. 2018 Aug 1;6:2172. Originally published 2017 Dec 22. [Version 2] doi: 10.12688/f1000research.13114.2 (PMC5990125; doi:10.12688/f1000research.13114.2)
Supplement: Supplementary file 4 [file f1000research-6-17227-s0003.tgz › 406c2df1-985b-47ab-a88d-7e94d8db368b.docx]

**Supplementary Table 1**

Parameters evaluated for each patient and included in clinical records. Not all parameters were evaluated in each visit.

| Age at onset | Cognitive impairment |
| --- | --- |
| Age at visit | Visual impairment |
| Routine visit | Visual field deficits/scotoma |
| Suspected Relapse | Diplopia (oculomotor nerve palsy) |
| Ambulation Index | Nystagmus |
| Nine-hole peg test (right) | Trigeminal nerve impairment |
| Nine-hole peg test (left) | Hemifacial spasm |
| PASAT | Dysarthria |
| Timed 25-Foot Walk | Dysphagia |
| Impairment in daily living activities | Facial nerve palsy |
| EDSS | Uper-limb motor deficit |
| Pyramidal Functions | Lower-limb motor deficit |
| Cerebellar Functions | Upper limb ataxia |
| Brainstem Functions | Lower limb ataxia |
| Sensory Functions | Dysesthesia |
| Bowel and Bladder Function | Hypoesthesia |
| Visual Function | Paresthesia |
| Cerebral (or Mental) Functions | Lhermitte's sign |
| Ambulation score | Urinary dysfunction |
| Fatigue | Bowel dysfunction |
| Mood disorders | Sexual dysfunction |
| Tremor | Ataxic gait |
| Headache | Paretic gait |
|  | Spastic gait |
