# Supplementary material for: Collaboration between a human group and artificial intelligence can improve prediction of multiple sclerosis course: a proof-of-principle study
Source: F1000Res. 2018 Aug 1;6:2172. Originally published 2017 Dec 22. [Version 2] doi: 10.12688/f1000research.13114.2 (PMC5990125; doi:10.12688/f1000research.13114.2)
Supplement: Peer review history

# Peer review: Referee response for version 1

**DOI:** 10.5256/f1000research.14226.r31371

**Published:** 2018-04-06

**Authors:** Bjoern Menze

**Affiliations:** 1 Department of Informatics, Technische Universität München, Munich, Germany

**General:**

 I think exploring how to fuse multiple expert opinions is a very interesting line of research in computer aided diagnostics. Here, the authors test how to make use of lay persons, and I would agree that there are many tasks when a (briefly trained) lay person or non-expert can contribute significantly to an analytical task.

 In the application here, I would be rather critical about this approach, though. For example, the authors write "through collective reasoning, or collective intelligence, groups of lay people may perform as well as experts." I would not agree, by any means. How would a lay person without training be able to distinguish, for example, a stroke related white matter hyper-intensity from an MS lesion? Or even a large MR artifact? Averaging will reduce variance in prediction, but the individual prediction itself has to be unbiased. In other words: the layman predictor has to be correct on average. But how would they possibly be in case they have no idea about how to read these data? Moreover, the authors point out that "studies with medical students show that working in pairs ameliorates diagnostic ability". Is this because of a better discussion of the decision? With two subjects it cannot be the power of large numbers that this study relies on.

 Instead of exploring how to fuse layman's decisions, I would recommend the authors to explore how to fuse decisions of different algorithms, or from neurologists of different training/seniority level, or decisions based on different sources.

**Technical:**

*Experimental setup and evaluation:* The authors describe a "leave-one patient-out" cross-validation as an innovation of their study. While this is a good approach, it is not new.

*Algorithm and training:* There are different classes - what is the distribution of those classes for the 84 patients? What is in the reports? Numbers? Free text? What features are input to the random forest algorithm? How many features at all? How did you train the algorithm (parameters "mtry", why 50 trees?) Without this information it is difficult to assess whether the performance of your random forest is bad (i.e., close to layman's predictions) because of an suboptimal training procedure, or because this is a hard problem indeed.

*Fusion rule:* (Described in the section "To compare the two sets... of the most consistent agent.") I don't understand what you do. How does summing a squared ranking lead to a prediction score? I assume you are using the normalized (and squared) ranking as a sort of weight? Why do you square the rankings? What happens when you use other non-linear transformations? Is there any way you illustrate the distributions so that we can follow your reasoning? How about presenting simple rules like averaging, or majority voting at least as a baseline we can compare against?

I have read this submission. I believe that I have an appropriate level of expertise to confirm that it is of an acceptable scientific standard, however I have significant reservations, as outlined above.

# Response

**Published:** 2018-07-26

**Authors:** Francesca Grassi

**Affiliations:** Sapienza University of Rome, Italy

First of all, thank you for taking the time to read our work and to give useful comments. We hope that our responses will clear your doubts. We list below the changes introduced in the new version, prompted by your observations. We hope that you agree with us that it is improved

*In the application here, I would be rather critical about this approach, though. For example, the authors write "through collective reasoning, or collective intelligence, groups of lay people may perform as well as experts." I would not agree, by any means. How would a lay person without training be able to distinguish, for example, a stroke related white matter hyper-intensity from an MS lesion? Or even a large MR artifact? Averaging will reduce variance in prediction, but the individual prediction itself has to be unbiased. In other words: the layman predictor has to be correct on average. But how would they possibly be in case they have no idea about how to read these data?*

 ANSWER

 Although your point of view is quite understandable, there is a large body of literature on the topic of collective intelligence. In the hope to overcome your skepticism on this point, we added some more references to published work on diagnostic crowdsourcing initiatives.

*Moreover, the authors point out that "studies with medical students show that working in pairs ameliorates diagnostic ability". Is this because of a better discussion of the decision? With two subjects it cannot be the power of large numbers that this study relies on.*

 ANSWER

 It is now better explained that the two quoted studies use different methods: real pairs in one, aggregated opinions in the other, yet both obtain better performances. Authors do not discuss the underlying processes, so we cannot indicate the real reason of a better performance.

*Instead of exploring how to fuse layman's decisions, I would recommend the authors to explore how to fuse decisions of different algorithms, or from neurologists of different training/seniority level, or decisions based on different sources*.

 ANSWER

 Thank you for your suggestion. Understanding that we have to deepen out study (now repeatedly stated throughout the paper) we have developed DiagnoShare to obtain the predictions of clinicians of different expertise and we are investigating the performance of algorithm combinations.

*Experimental setup and evaluation: The authors describe a "leave-one patient-out" cross-validation as an innovation of their study. While this is a good approach, it is not new.*

 ANSWER

 Thank you for your observation. Indeed, it is better to define our approach as a modified leave-one-out method. It is modified, because we not only left one patient out, we also included only one record for each of the remaining patients.

*Algorithm and training:* *There are different classes - what is the distribution of those classes for the 84 patients? What is in the reports? Numbers? Free text? What features are input to the random forest algorithm? How many features at all? How did you train the algorithm (parameters "mtry", why 50 trees?) Without this information it is difficult to assess whether the performance of your random forest is bad (i.e., close to layman's predictions) because of an suboptimal training procedure, or because this is a hard problem indeed*

 ANSWER

 Thank you for pointing out that this part of the paper required clarification. As now better emphasized in the text, in this proof-of-concept work we considered only patients that actually transitioned to the SP phase, so there is a unique class of patients. Features input to the RF algorithm are listed in Supplementary Table 1. We added a statement to declare what types of numerical values we used in the work. The results presented show that the RF algorithm performs better than layman, as its performance is however better than that of individual medical students, that are not quite laymen, although not experts as well. In any case, the focus of the paper is not on the goodness of the algorithm, but on the value of combining different approaches to the prediction problem, which indeed has been resisting solution for many years of medical analysis.

*Fusion rule:* *(Described in the section "To compare the two sets... of the most consistent agent.") I don't understand what you do. How does summing a squared ranking lead to a prediction score? I assume you are using the normalized (and squared) ranking as a sort of weight? Why do you square the rankings? What happens when you use other non-linear transformations? Is there any way you illustrate the distributions so that we can follow your reasoning? How about presenting simple rules like averaging, or majority voting at least as a baseline we can compare against?*

 ANSWER

 We agree with you that, indeed, this point is complex and we try a different explanation, hoping that it is clearer. First of all, ranking is inherent to building a ROC curve. Since we have only two agents (humans and RF algorithm), we cannot use a majority rule, we can only perform an average (linear or weighted) of the scores. For any clinical record, the final forecast is the average of "unitary predictions" by multiple individuals or decision trees. If "unitary predictions" of one agent are highly concordant, it means that the prediction is quite obvious for the agent, suggesting that is more probably correct than others. We ranked forecasts on clinical records in order of concordance of "unitary predictions" and emphasized the value of agreement by squaring the ranks.

 In line with other pieces of research, this weighted average performed better than linear averaging, as stated in the paper

# Peer review: Referee response for version 1

**DOI:** 10.5256/f1000research.14226.r31369

**Published:** 2018-03-21

**Authors:** Roger Tam

**Affiliations:** 1 Department of Radiology and MS/MRI Research Group, University of British Columbia, Vancouver, BC, Canada

This is an interesting and clearly written article on using a machine learning method (random forests) and medical students to form "hybrid" predictions of disease progression in MS, specifically the conversion from RRMS to SPMS. The article claims that the results are a proof-of-principle that combining machine learning and human predictions is better than either approach alone. 

 The main strengths of the article are its clear writing, the reproducbility of the experiments, the clinical importance of the application, and topical nature of the subject, as machine learning for clinical prediction is such a hot topic that integration with the clinical workflow is a critical area of study.

 The main limitations of the article are that only clinical parameters were used to perform the predictions, and the longitudinal nature of the data was not used to its full benefit. To realize the potential of machine learning for MS prediction, imaging parameters should be included (there is good literature on MS prediction using imaging), and examining changes over time is important for both machine (eg, using recurrent networks) and human raters (examining clinical changes over multiple time points). The article places some importance on having the computer and humans using the same set of input parameters, but I do not feel that this is warranted; the data should be selected to be most appropriate for each approach.

 Given the above limitations, it is difficult to generalize the findings to say that hybrid predictions are better than either machine learning or humans. This could be true, and the article provides some support for that, but more work needs to be done to provide strong evidence.

I have read this submission. I believe that I have an appropriate level of expertise to confirm that it is of an acceptable scientific standard, however I have significant reservations, as outlined above.

# Response

**Published:** 2018-07-26

**Authors:** Francesca Grassi

**Affiliations:** Sapienza University of Rome, Italy

First of all, thank you for taking the time to read our work and to give useful comments. We hope that our responses will clear your doubts. We list below the changes introduced in the new version, prompted by your observations. We hope that you agree with us that it is improved.

*The main limitations of the article are that only clinical parameters were used to perform the predictions, and the longitudinal nature of the data was not used to its full benefit. To realize the potential of machine learning for MS prediction, imaging parameters should be included (there is good literature on MS prediction using imaging), and examining changes over time is important for both machine (eg, using recurrent networks) and human raters (examining clinical changes over multiple time points). The article places some importance on having the computer and humans using the same set of input parameters, but I do not feel that this is warranted; the data should be selected to be most appropriate for each approach.*

 ANSWER

 We agree with you that many other approaches could be used. As now stated in the text, we chose to explore predictions done using only clinical data, available to all neurologists, which have recently been independently demonstrated to have good predictive value (Goodin et al., 2018; reference added to the paper). Imaging data performed in real-world clinical settings do not have the standardization required for predictions either by experts or algorithms. However, future studies aimed at confirming this proof-of-principle, initial work will surely consider different options.

*Given the above limitations, it is difficult to generalize the findings to say that hybrid predictions are better than either machine learning or humans. This could be true, and the article provides some support for that, but more work needs to be done to provide strong evidence.*

 ANSWER

 We completely agree with you that this is a preliminary, proof-of-concept work. We state it more clearly in the Discussion

# Peer review: Referee response for version 1

**DOI:** 10.5256/f1000research.14226.r30349

**Published:** 2018-02-26

**Authors:** Bruno Bonetti

**Affiliations:** 1 USD Stroke Unit, DAI di Neuroscienze, Azienda Ospedaliera Universitaria Integrata, Verona, Italy

The manuscript is interesting and intriguing, since it opens new possibilities in MS prognosis combining human expertise and "artificial intelligence". I do not understand why medical students have been chosen instead of (young) neurologists who may have additional skills in the specific task. Apart from this aspect, the manuscript is well written and easy to follow. Deserves publication.

I have read this submission. I believe that I have an appropriate level of expertise to confirm that it is of an acceptable scientific standard.

# Response

**Published:** 2018-03-01

**Authors:** Francesca Grassi

**Affiliations:** Sapienza University of Rome, Italy

Thank you very much for reviewing our paper.

In this proof-of-concept study, we chose to work with medical students instead of neurologists because we wanted to test if even a group of relatively uneducated people can enhance the predictive ability of machine learning algorithms, which is now well established.

We agree with you that the next step is to obtain predictions by neurologists and other medical doctors, and in fact we set up the platform DiagnoShare ( http://www.phys.uniroma1.it/diagnoshare) to extend the study.

Hopefully, we can soon extend this work with a final study
